# 713 Mortality Benefit After Addition of Mid-level Support in Burn Intensive Care Unit

**DOI:** 10.1093/jbcr/irac012.267

**Published:** 2022-03-23

**Authors:** Jamie L Hollowell, Felicia Williams, Daniel Blandon-Hendrix, Booker King, Lori Chrisco, Eli Maxwell, Rabia Nizamani

**Affiliations:** UNC Jaycee Burn Center, Hurdle Mills, North Carolina; UNC Jaycee Burn Center, Chapel Hill, North Carolina; UNC Jaycee Burn Center, Chapel Hill, North Carolina; UNC Jaycee Burn Center, Chapel Hill, North Carolina; North Carolina Jaycee Burn Center, Chapel Hill, North Carolina; UNC Jaycee Burn Center, Chapel Hill, North Carolina; UNC Jaycee Burn Center, Chapel Hill, North Carolina

## Abstract

**Introduction:**

Burn Intensive Care Units (BICU)s are resource-heavy and labor-intensive units with very sick patients. The removal of burns as a requirement from the surgical curriculum has decreased the number of rotating surgical trainees, but did not impact patient care needs. Our unit adopted an Advanced Practice Provider (APP) service model in fiscal year 2018 to provide consistent standardized clinical care, with surgical trainees rotating monthly, to mitigate the loss of residents over time. We aimed to critically evaluate the impact of an APP run BICU on mortality and quality improvement initiatives.

**Methods:**

Patients were identified using Institutional Burn Center registry, and linked to the clinical and administrative data. All patients admitted to the BICU between July 1, 2016 and June 30, 2020 were eligible for inclusion. All central line associated blood stream infections (CLABSI), catheter associated urinary tract infections (CAUTI), ventilator associated pneumonias (VAP) and mortality rates were compared. Demographics, length of stay (LOS), co-morbid conditions and mortality were evaluated. Statistical analysis was performed with Students’ t-test, and chi-squared tests. Significance was accepted as p< 0.05.

**Results:**

There were no significant differences in admission rates over the study period. The number of CLABSIs significantly decreased each year (15 (2017), 6 (2018), 5 (2019), 3 (2020)). The number of CAUTIs significantly decreased ((13 (2017), 6 (2018), 1 (2019), 3 (2020)). The number of VAPs significantly decreased ((15(2017), 12 (2018), 7 (2019), 3 (2020)). Mortality was unchanged from 2017-2019 but significantly decreased in 2020 ((2.2% (2017), 2.4% (2018), 2.5% (2019), 0.9% (2020)).

**Conclusions:**

There were no significant differences in admission rates over the study period. The number of CLABSIs significantly decreased each year (15 (2017), 6 (2018), 5 (2019), 3 (2020)). The number of CAUTIs significantly decreased ((13 (2017), 6 (2018), 1 (2019), 3 (2020)). The number of VAPs significantly decreased ((15(2017), 12 (2018), 7 (2019), 3 (2020)). Mortality was unchanged from 2017-2019 but significantly decreased in 2020 ((2.2% (2017), 2.4% (2018), 2.5% (2019), 0.9% (2020)).

